# In vivo estimation of passive biomechanical properties of human myocardium

**DOI:** 10.1007/s11517-017-1768-x

**Published:** 2018-02-26

**Authors:** Arnab Palit, Sunil K. Bhudia, Theodoros N. Arvanitis, Glen A. Turley, Mark A. Williams

**Affiliations:** 1 WMG, The University of Warwick, Coventry, CV4 7AL UK; 2 Institute of Digital Healthcare, WMG, The University of Warwick, Coventry, UK; 3 University Hospitals Coventry and Warwickshire, Coventry, UK

**Keywords:** Ventricular diastolic mechanics, Finite element, Parameter estimation, Normal human subjects, Ventricular geometry, Fibre structure

## Abstract

Identification of in vivo passive biomechanical properties of healthy human myocardium from regular clinical data is essential for subject-specific modelling of left ventricle (LV). In this work, myocardium was defined by Holzapfel-Ogden constitutive law. Therefore, the objectives of the study were (a) to estimate the ranges of the constitutive parameters for healthy human myocardium using non-invasive routine clinical data, and (b) to investigate the effect of geometry, LV end-diastolic pressure (EDP) and fibre orientations on estimated values. In order to avoid invasive measurements and additional scans, LV cavity volume, measured from routine MRI, and empirical pressure-normalised-volume relation (Klotz-curve) were used as clinical data. Finite element modelling, response surface method and genetic algorithm were used to inversely estimate the constitutive parameters. Due to the ill-posed nature of the inverse optimisation problem, the myocardial properties was extracted by identifying the ranges of the parameters, instead of finding unique values. Additional sensitivity studies were carried out to identify the effect of LV EDP, fibre orientation and geometry on estimated parameters. Although uniqueness of the solution cannot be achieved, the normal ranges of the parameters produced similar mechanical responses within the physiological ranges. These information could be used in future computational studies for designing heart failure treatments.

**Figure:**
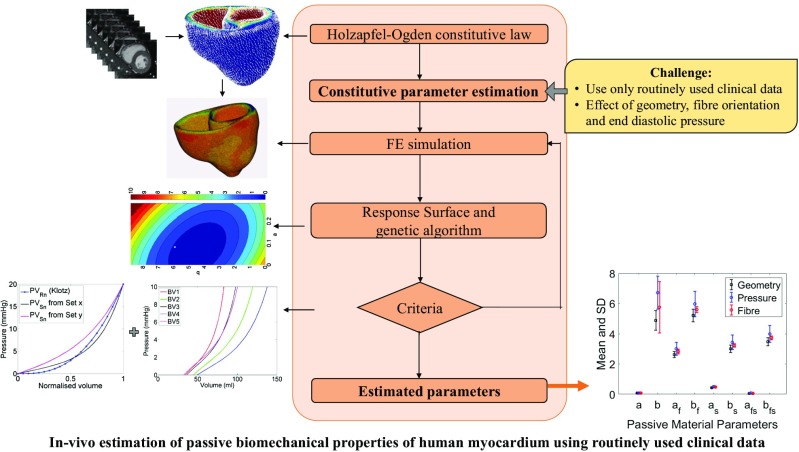
Graphical abstract

Graphical abstract

## Introduction

Local myocardial wall stress is hypothesised to be responsible for many cardiac mechanisms, including ventricular remodelling, which is frequently associated with heart failure (HF). However, stress in the left ventricle (LV) cannot be measured directly [20]. Finite element (FE) method, in combination with advanced simulation tools and new cardiac imaging modalities can be used to analyse LV wall stress–strain distribution for providing a greater insight of the physiology of normal subjects and HF patients, and thereby, predict their responses to medical and surgical interventions [3, 6, 13, 14, 19, 25, 38, 39, 48, 51]. In such models, it is essential to accurately use in vivo passive biomechanical properties of the human myocardium in order to mimic the cardiac mechanics properly. Otherwise, the stress-strain prediction would be over or under estimated which will lead to inaccurate diagnostic information for surgical operation [61].

Traditionally, ex vivo mechanical testing was carried out on myocardial tissue, harvested from a specific heart, to identify its properties. Results from the biaxial tests of canine myocardium [7, 21, 31, 63] and simple shear test of pig [9] and human [43, 44] myocardium clearly exhibited its orthotropic behaviour. These experimental data not only provided the understanding to define the constitutive laws for the myocardium material, but also helped in determining the values of the parameters [41, 42]. These traditional methods involve invasive ex vivo procedures and result in the destruction of the myocardial tissues. These methods are therefore not ideal for in vivo clinical measurement [8]. Moreover, ex vivo experiments using cadaver hearts may not be the true representative of the in vivo passive properties of the heart as these hearts do not represent real-life conditions due to the lack of homeostasis [56].

FE modelling, in combination with cardiac magnetic resonance imaging (CMRI), was carried out to estimate passive myocardial properties in a non-invasive manner [12, 13, 50, 56, 60–62]. Other work was accomplished to assess the passive material properties in isolated hearts with FE analysis [2, 10, 29, 32, 33, 45]. Most of them used Fung-type transversely isotropic law [5, 16, 17, 22]. In contrast, the orthotropic behaviour of myocardium was evident from the simple shear test of pig’s [9] and human myocardium [44]. Moreover, Schmid, O’Callaghan [42] reported that a transversely isotropic law would not be suitable for modelling orthotropic responses of passive myocardium under simple shear tests. Modified Fung-type law [1, 49] and pole-zero law [30, 45] were introduced to incorporate myocardial material orthotropy. However, in all the Fung-type material models, the material parameters were merely used as weighting factors, rather than providing any physical importance [15]. Besides, some of the material parameters were highly correlated in Fung-type and pole-zero law [15, 40, 45, 52]. Recently, Holzapfel and Ogden [18] developed a constitutive law that considered the locally orthotropic tissue architecture and the parameters of this model were closely related to the characteristic micro-structure of myocardium. In literature, the parameters of the material model were fitted to match the simple shear test results from pig myocardium [9] to define fully orthotropic nature. The values of these fitted parameters (Table 2) resulted in too stiff stress–strain relation in patient-specific model, and therefore, unable to produce measured end-diastolic volume (EDV) of LV within the physiological LV end-diastolic pressure (EDP) [3, 11, 52]. Table 1 summarised the methods used to estimate the myocardial properties and limitation in research gap which is addressed in this paper.

**Table 1:** A brief overview of the methods to estimate constitutive parameters of myocardium and current research gap

	Using mechanical test results of excised myocardium	Isolated heart with FE analysis	In vivo estimation using FE (animal myocardium)	In vivo estimation using FE (human myocardium)
Fung-type transversely isotropy	Schmid et al. (2006) Schmid et al. (2008)	Omens et al. (1993) Augenstein et al. (2005) Augenstein et al. (2006) Nair et al. (2007)	Walker et al. (2005) Wang et al. (2009)	Xi et al. (2011a) Xi et al. (2011b) Xi et al. (2013) Genet et al. (2014)
Pole-zero orthotropic		Stevens et al. (2003) (Remme et al., 2004)		
Fung-type orthotropic	Schmid et al. (2006) Schmid et al. (2008)			
Holzapfel-Ogden orthotropic	Holzapfel and Ogden (2009) Göktepe et al. (2011) Wang et al. (2013a) Eriksson et al. (2013)			Research gap

An inverse estimation method, which is typically formulated as a non-linear optimisation problem to minimise the differences in the measurements with respect to the unknown parameters, was used to quantify passive properties of human myocardium using Holzapfel-Ogden material law. Due to the highly non-linear nature of the inverse optimisation problem, large design space and correlation amongst the material parameters, it is non-trivial to inversely estimate those parameters accurately and uniquely using limited clinical data. The trade-off is that while fewer clinical data make the inverse problem more ill-posed, requirement of more subject-specific data (i.e. MRI tagging and in-vivo ventricular pressure) leads to more complex and invasive clinical measurements with longer processing times, which is not always possible. Therefore, one of the major challenges, addressed in this study, was to extract the human myocardial properties instead of finding unique solution using non-invasive routinely used clinical data rather than using invasive and computationally expensive clinical data. Therefore, the study proposed a method to achieve the following objectives: (a) to estimate the normal ranges of Holzapfel-Ogden parameters for healthy human myocardium using routinely used non-invasive clinical data, and (b) to explore the effect of geometry, fibre orientation and EDP on the estimation of passive parameters.

## Methods

### FE modelling of ventricle

**Fig. 1:**
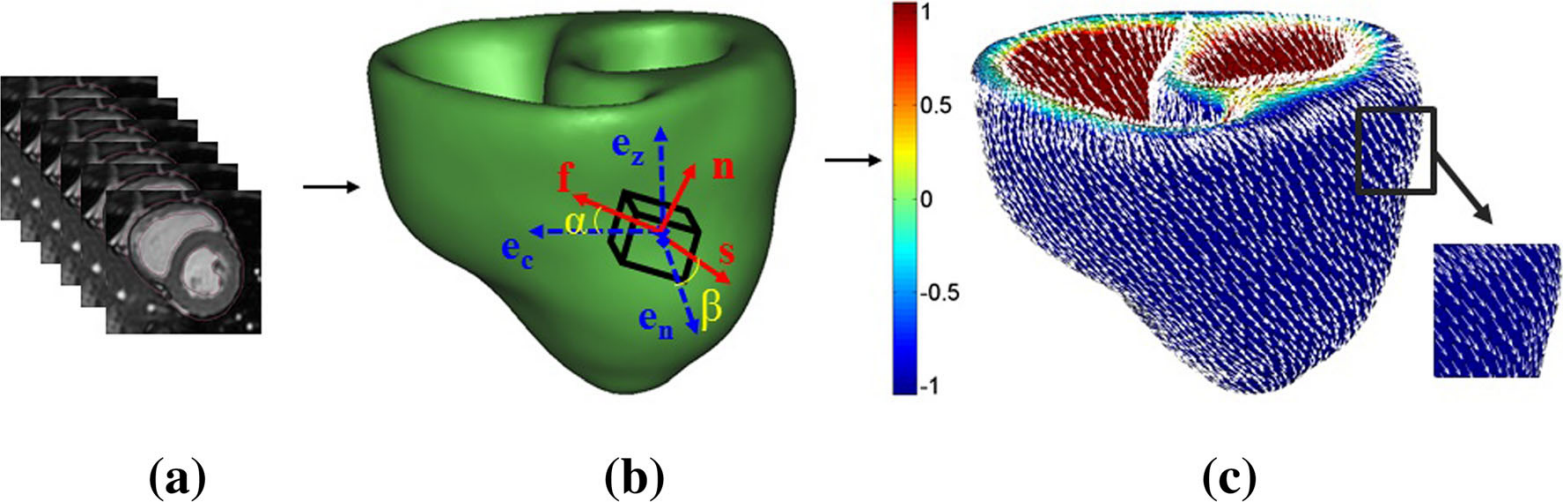
Construction of BV geometry from CMRI and assign fibre orientation on the constructed BV mesh geometry using LDRF algorithm. **a** Short-axis CMRI. **b** BV mesh geometry with fibre-sheet directions and local cardiac coordinate. **c** Assigned fibre orientation

**Fig. 2:**
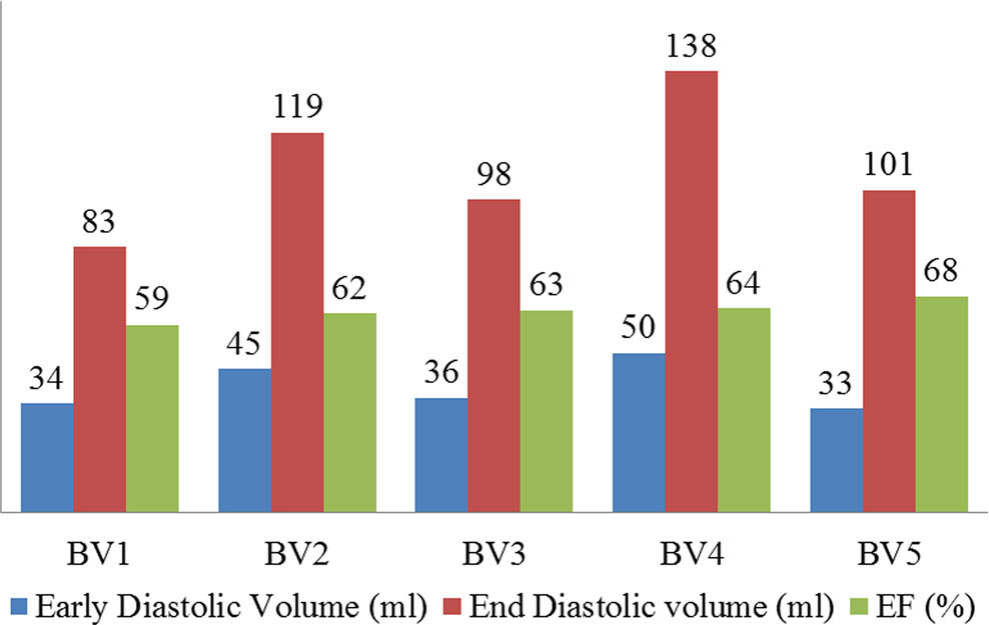
Early diastolic volume (ErDv), end diastolic volume (EDV) and ejection fraction (EF) extracted from CMRI for five healthy hearts (BV1 to BV5)

In the present study, ECG gated, breath hold, steady-state free precession (SSFP) cine CMRI was used to capture the images of five normal human ventricles at UHCW, UK. BSREC ethics approval (REGO-2012-032) and patients’ consent were obtained to carry out the research on anonymised human data. Mimics and 3-matic (Materialise, Belgium) were used to construct the bi-ventricular (BV) mesh geometry form CMRI as detailed in Palit, Turley [37] (Fig. 1a, b). The early diastolic volume (ErDV), end diastolic volume (EDV), end systolic volume (ESV) and, finally the ejection fraction (EF) for each subject were calculated subsequently (Fig. 2). Each BV mesh geometry composed of at least 740,000 linear tetrahedral elements (tet4) to achieve accurate results as identified from the mesh convergence study by Palit, Bhudia [35]. Details of the MRI scanning protocol and demographic information of the subjects are enclosed in Appendix 1. Myocardial fibre structure was implemented by Laplace-Dirichlet-Region growing-FEM (LDRF) algorithm [37] (Fig. 1c). Based on previous histological studies [1, 46], the fibre direction was defined by a linear variation of helix angle (α) from − ;70° in the epicardium and right ventricular (RV) septal endocardium to almost 0° in the mid-wall to + ;70° at endocardium and right ventricular free wall endocardium for all five ventricles. The Holzapfel-Ogden material law used here has been described extensively in Holzapfel and Ogden [18] and Göktepe, Acharya [15]. The key points are summarised here and details are given in Appendix 2. There are total of eight non-negative material parameters (*a*, *b*, *a*_*f*_, *b*_*f*_, *a*_*s*_, *b*_*s*_, *a*_*fs*_, *b*_*fs*_)in the strain energy function along with an extra bulk modulus (*K*) as shown in Eq. (1).

1 $$ {\displaystyle \begin{array}{l}\varPsi =K\left(\frac{J^2- 1}{2}- lnJ\right)+\frac{a}{2b}\;\exp \left[b\left({\overline{I}}_1- 3\right)\right]\;\\ {}\kern1.08em +\sum \limits_{i=f,s}\frac{a_i}{2{b}_i}\;\left\{\exp \left[{b}_i{\left({\overline{I}}_{4i}- 1\right)}^2\right]- 1\right\}+\frac{a_{fs}}{2{b}_{fs}}\left\{\exp \left[{b}_{fs}{\left({\overline{I}}_{8fs}\right)}^2\right]- 1\right\}\end{array}} $$

where *a* and *b* parameters represent the response of isotropic ground matrix; *a*_*f*_ and *b*_*f*_ characterise the response of myocardial fibres; *a*_*s*_ and *b*_*s*_ denote the contribution of sheet; and *a*_*8fs*_ and *b*_*8fs*_ account for the shear effects in the sheet plane. All *a*_*i*_ (i = f, s, fs) and  *a* represent the dimension of stress in kPa and the unit-less parameter b_i_ (i = f, s, fs) and  *b* represent the non-linear behaviour of the corresponding structure. The bulk modulus (*K*) works as a penalty parameter for imposing incompressibility.

Holzapfel-Ogden constitutive law was implemented using a user-defined subroutine ‘Hypela2’ in MSC-Marc (MSC Software Corporation, USA) [35] . The early diastole (ErD) BV mesh geometry was constructed from the CMRI. Early diastole was assumed as initial stress-free configuration since the ventricular pressure is lowest at this point, and consequently, wall stress is minimum [12, 13, 35, 36, 47, 49, 57, 59]. Due to the unavailability of subject-specific ventricular pressures requiring invasive measurements, we were compelled to assume the LV EDP as 10 mmHg [13, 26, 27, 52]. One third of the LV blood pressure was applied on the RV endocardium [15, 35]. The longitudinal movement of the base nodes and the circumferential displacement of epicardial wall at the base were suppressed in order to avoid undesirable rigid body displacement [11, 13, 55, 58]. The rest of the ventricular wall including apex was left free [11, 13, 55, 58].

### Investigation strategy

As outlined by Xi, Lamata [61] in the context of inverse estimation of material parameters, multiple parameter sets are able to reproduce similar end-diastolic deformation states. Therefore, few strategic and logical assumptions were made as follows:

#### Define the range of the parameters

From the definition of the material law, all the eight parameters are positive real number (i.e. *a*, *b*, *a*_*f*_, *b*_*f*_, *a*_*s*_, *b*_*s*_, *a*_*fs*_, *b*_*fs*_ > 0). On the other hand, it was reported that the human myocardium is less stiff than pig myocardium [13, 44]. Therefore, the shear stress for each shear mode [18] should be lower for human myocardium compared to pig myocardium. Thus, the maximum value of each parameter should not be higher than the value estimated from the shear stress data of pig myocardium (Table 2). In this study, the average values, shown in Table 2, was considered as the maximum value for the respective parameters. Therefore,

2 $$ \left.\begin{array}{l}{a}^{\mathrm{max}}= 0.28,\operatorname{}{b}^{\mathrm{max}}= 8.82,\\ {}{a_f}^{\mathrm{max}}= 18.1,\kern0.5em \operatorname{}{b_f}^{\mathrm{max}}= 16.5,\\ {}{a_s}^{\mathrm{max}}= 3.0,\operatorname{}{b_s}^{\mathrm{max}}= 9.5,\\ {}{a_{8fs}}^{\mathrm{max}}= 0.4,\operatorname{}{b_{8fs}}^{\mathrm{max}}= 10.9\ \end{array}\right\} $$

**Table 2:** Values of Holzapfel-Ogden passive material parameters used in existing literature

Articles	Passive material parameters							
	a (KPa)	b	a_f_ (KPa)	b_f_	a_s_ (KPa)	b_s_	a_fs_ (KPa)	b_fs_
Holzapfel and Ogden (2009)	0.059	8.023	18.472	16.026	2.481	11.120	0.216	11.436
Göktepe et al. (2011)	0.496	7.209	15.193	20.147	3.283	11.176	0.662	9.466
Wang et al. (2013a)	0.236	10.81	20.037	14.154	3.724	5.164	0.411	11.3
Eriksson et al. (2013)	0.333	9.242	18.535	15.972	2.564	10.446	0.417	11.602
Average (approximated)	0.28	8.82	18.1	16.5	3	9.5	0.4	10.9

#### Reduce the design space

Without changing the constitutive law, the design space of the problem was reduced by selecting fewer parameters for the inverse estimation using similar method described in Xi, Lamata [61]. It was observed from the experiment of pig myocardium that the order of shear responses in six shear modes would follow as ${\sigma}^{(fs)}>{\sigma}^{(fn)}>{\sigma}^{(sf)}>{\sigma}^{(sn)}>{\sigma}^{(nf)},{\sigma}^{\left(\mathrm{ns}\right)}$ (where *ij* denoted the shear response in *j* direction of the plane containing *i* direction and *i* ≠ *j* ∈ {*f*, *s*, *n*}) [9, 18]. The analytical expressions of these shear stress modes, used to fit the constitutive parameters to match the experimental data, are shown from Eq. (3) to Eq. (8) [15].

3 $$ {\sigma}^{(fs)}=\gamma\;a\;\exp \left[{\gamma}^2\;b\right]+ 2\;{\gamma}^3\;{a}_f\;\exp \left[{\gamma}^4\;{b}_f\right]+\gamma\;{a}_{fs}\;\exp \left[{\gamma}^2\;{b}_{fs}\right] $$

4 $$ {\sigma}^{(fn)}=\gamma\;a\;\exp \left[{\gamma}^2\;b\right]+ 2\;{\gamma}^3\;{a}_f\;\exp \left[{\gamma}^4\;{b}_f\right] $$

5 $$ {\sigma}^{(sf)}=\gamma\;a\;\exp \left[{\gamma}^2\;b\right]+ 2\;{\gamma}^3\;{a}_s\;\exp \left[{\gamma}^4\;{b}_s\right]+\gamma\;{a}_{fs}\;\exp \left[{\gamma}^2\;{b}_{fs}\right] $$

6 $$ {\sigma}^{(sn)}=\gamma\;a\;\exp \left[{\gamma}^2\;b\right]+ 2\;{\gamma}^3\;{a}_s\;\exp \left[{\gamma}^4\;{b}_s\right] $$

7 $$ {\sigma}^{(nf)}=\gamma\;a\;\exp \left[{\gamma}^2\;b\right] $$

8 $$ {\sigma}^{(ns)}=\gamma\;a\;\exp \left[{\gamma}^2\;b\right] $$

where *γ* = amount of shear. From Eq. (3) to (8), it is observed that the order of shear stiffness mode does not depend on the parameters *a* and *b*. The last six parameters are only responsible for maintaining the ordering as is evident from Eq. (3) to (6). The ordering can always be maintained if the ratios amongst *a*_*i*_ (and *b*_*i*_) are kept same (i.e. if *a*_*f*_ *: a*_*s*_ *: a*_*fs*_ = constant and *b*_*f*_ *: b*_*s*_ *: b*_*fs*_ = constant). Therefore, all the *a*_*i*_ and *b*_*i*_ (where *i* = *f*, *s*, *fs*) should be divided by *Ka* and *Kb*, respectively, so that Eqs. (9) and (10) are maintained. *Ka* and *Kb* are positive real number.

9 $$ \frac{a_f}{Ka}=\frac{a_s}{Ka}=\frac{a_{fs}}{Ka}=\mathrm{constant} $$

10 $$ \frac{b_f}{Kb}=\frac{b_s}{Kb}=\frac{b_{fs}}{Kb}=\mathrm{constant} $$

Thus, only four independent parameters (*a*, *b*, *Ka*, *Kb*) were required for inverse estimation to maintain the observed shear stiffness ordering [9]. The last six material parameters for human myocardium were then calculated using Eqs. (2), (9) and (10), as

11 $$ {a}_f^{\mathrm{human}}=\frac{a_f^{\mathrm{max}}}{Ka}=\frac{18.1}{Ka};{a}_s^{\mathrm{human}}=\frac{a_s^{\mathrm{max}}}{Ka}=\frac{3}{Ka};\kern1em {a}_{fs}^{\mathrm{human}}=\frac{a_{fs}^{\mathrm{max}}}{Ka}=\frac{0.4}{Ka} $$

12 $$ {b}_f^{\mathrm{human}}=\frac{a_f^{\mathrm{max}}}{Kb}=\frac{16.5}{Kb};{b}_s^{\mathrm{human}}=\frac{a_s^{\mathrm{max}}}{Kb}=\frac{9.5}{Kb};{b}_{fs}^{\mathrm{human}}=\frac{a_{fs}^{\mathrm{max}}}{Kb}=\frac{10.9}{Kb} $$

If it was assumed that the *a*_*f*_ and *b*_*f*_ values for human myocardium would not be less than 1, the maximum value of *Ka* and *Kb* should not be greater than 18.1 and 16.5, respectively. On the other hand, the minimum value of *Ka* and *Kb* should not be less than 1 as ${a}_i^{\mathrm{human}}<={a}_i^{\mathrm{max}}$ and ${b}_i^{\mathrm{human}}<={b}_i^{\mathrm{max}}$ where *i* = *f*, *s*, *fs*. The explicit definitions of the ranges are shown from Eqs. (17) to (20).

#### Problem formulation

**Fig. 3:**
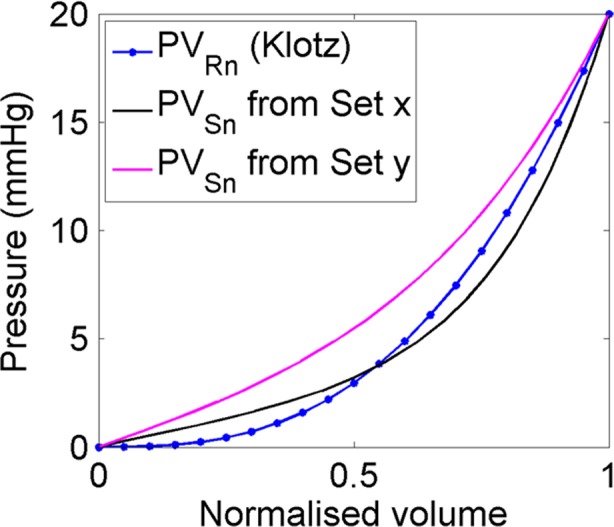
An exemplary graphical representation of the closeness between the empirical Klotz curve in 20 mmHg scales (PV_Rn_) and model predicted pressure-normalised volume (PV_*Sn*_) curve. ‘Set *x*’ predicted closer PV_Sn_ relation compare to ‘set *y*’ (closeness values, calculated using Eq. (16), for set *x* and set *y* are 1.5 and 0.16, respectively). Each set is a unique combination of the four parameters within each respective range

The main goal of the optimisation problem was to estimate the values of four parameters (*a*, *b*, *Ka*, *Kb*) so that the passive inflation of LV could produce ‘real’ LV EDV at EDP where real referred to MRI measured volume. Therefore, the objective function was to minimise the differences between the FE predicted and real LV cavity volume (Eq. (15)). There would be various combinations of four parameters for each given set of LV EDV and EDP, although all the combinations might not provide the correct end-diastolic pressure volume relation (EDPVR) of LV. Moreover, subject-specific EDPVR can only be measured invasively which is not a routine clinical practice. Thus, the Klotz empirical relation [23] was used in addition to matching the measured LV EDV at EDP. The normalised volume can be calculated with respect to any pressure point (e.g. 10, 15, 20 or 30 mmHg) [23]. However, pressure point less than 15 mmHg was not preferred because the pressure-volume prediction was better when it was predicted by a point having EDP of more than 15 mmHg [23]. Therefore, the normalised LV volume in this study was calculated with respect to 20 mmHg scale as defined by Eq. (13), and thereafter, the model predicted pressure-normalised volume (PV_Sn_) was measured.

13 $$ {EDV}_n=\frac{\left(V-{V}_0\right)}{\left({V}_{20}-{V}_0\right)} $$

where *V*_0_ and *V*_20_ are LV volume at 0 and 20 mmHg, respectively, *V* is the volume at a certain LV EDP. The empirical Klotz-curve (PV_Rn_) was then derived by varying EDV_*n*_ from 0 to 1 in Eq. (14).

14 $$ EDP= 20\times {EDV}_n^{B_{\boldsymbol{n}}} $$

where EDV_*n*_ is normalised volume, and *B*_n_ = 2.76. The closeness of the two curves (model predicted PV_Sn_ and PV_Rn_, i.e. Klotz curve) was calculated using Eq. (16), which was solved by using ‘trapz’ function in MATLAB (Fig. 3). The closeness of the two curves (Eq. (16)) was, therefore, used as a constraint for the optimisation problem. The maximum value of the constraint (Eq. (16)) was assumed from the initial feasibility study. Hence, the optimisation problem formulation was defined as follows:

15 $$ {\displaystyle \begin{array}{l}\operatorname{Min}i\mathrm{mise}\ {F}_{\mathrm{obj}}\;\left(a,b, Ka, Kb\right)\operatorname{}\\ {}\mathrm{where}\operatorname{}{F}_{\mathrm{obj}}= abs\left(\;{V}_{\mathrm{Rn}}-{V}_{\mathrm{Sn}}\right)\end{array}} $$

Subject to,

16 $$ 0<\mathrm{abs}\;\left[\underset{0}{\overset{1}{\int }}f\left({PV}_{Rn}\right)\; dv-\underset{0}{\overset{1}{\int }}f\left({PV}_{\mathrm{Sn}}\right)\; dv\;\right]< 0.3 $$

17 $$ a\in \left(0,0. 28\right) $$

18 $$ b\in \left(0, 8.82\right) $$

19 $$ Ka\in \left(1,18.1\right) $$

20 $$ Kb\in \left(1,16.5\right) $$

where

*V*_*Rn*_: Real (MRI measured) normalised volume = 100* (EDV_*R*_−ErDV_*R*_)/EDV_*R*_,
*V*_*Sn*_: Simulated (FE generated) normalised volume = 100*(*EDV*_*S*_*−ErDV*_*R*_)*/EDV*_*S*_
*EDV*_*R*_: Real end diastolic volume measured from CMRI,
*ErDV*_*R*_ : Real early diastolic volume measured from CMRI,
*EDV*_*S*_ : End diastolic volume generated from simulation,
*PV*_*Rn*_: Empirical Klotz curve in 20 mmHg pressure scale (Eq. (14)),
*PV*_*Sn*_: Pressure normalised volume curve generated from FE results
*dv*: Infinitesimally small volume
*abs*: Absolute value

#### Solving the optimisation problem

**Fig. 4:**
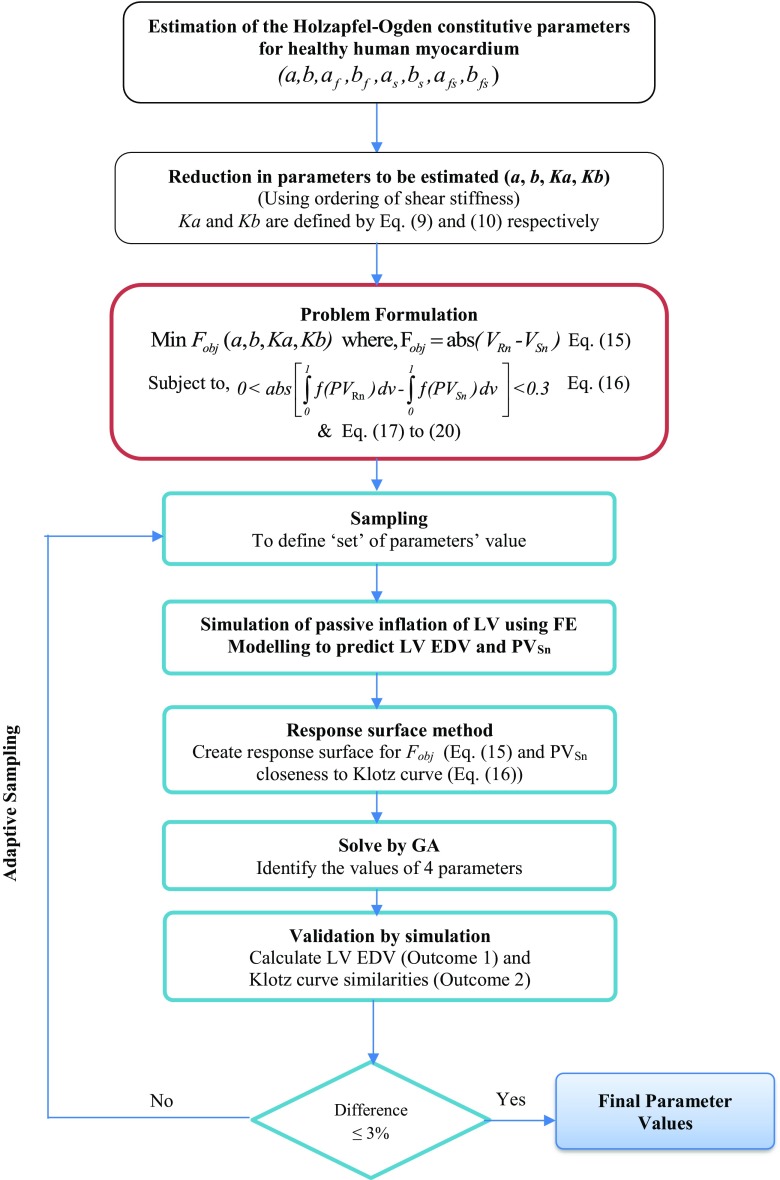
Flow chart of the proposed inverse optimisation procedure to estimate the values of the Holzapfel-Ogden constitutive parameters for healthy human myocardium

Initial sampling was carried out to create 50 ‘sets’ of parameters using the range of parameters defined in Eqs. (17)–(20). Each set is a unique combination of the four parameters within their respective ranges (Eq. (17)–(20)). In this study, Latin hypercube sampling (LHS) was used and was implemented in a customised script in MATLAB to generate uniformly distributed sets of parameters. Simulation of passive inflation using each set was then carried out using FE modelling and boundary conditions mentioned in Section 2.1. Two outputs (LV EDV and closeness of the PV_Sn_ to Klotz curve (PV_Rn_), i.e. Eq. (16)) were measured from each simulation. Empirical model of the two responses (Eqs. (15) and (16)) with respect to four independent material parameters were developed using response surface method (RSM). Genetic algorithm (GA) was then used to solve the optimisation problem to identify the optimal set of parameters. MATLAB GA function with all the default parameter values was used in this study. The optimised parameters from GA were used in FE model again, and the EDV and Klotz curve closeness was measured. If the absolute differences between GA results and simulation results were greater than 3%, the new set with its simulation results was included in parameters set to modify the response surface. The process continued until the simulation results matched with GA results with in ± 3% range. A flow chart of the proposed inverse optimisation procedure is depicted in Fig. 4.

## Results

### In vivo estimations of the passive properties

**Fig. 5:**
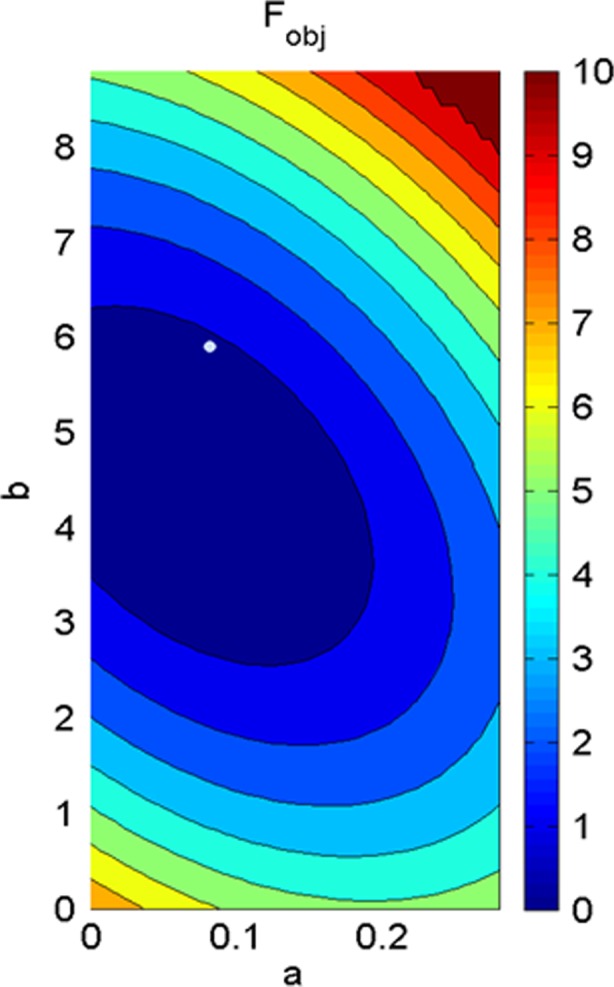
Landscape of objective function (*F*_obj_) related to the parameters *a* and *b* for BV1 when LV EDP was assumed as 10 mmHg

Table 3 shows the values of four material parameters (*a*, *b*, *Ka*, *Kb*) for five human BVs using the proposed inverse optimisation procedure (Fig. 4). The six material parameters corresponding to fibre-sheet (*a*_*f*_, *b*_*f*_, *a*_*s*_, *b*_*s*_, *a*_*fs*_, *b*_*fs*_) were then derived using Eqs. (11) and (12). The values of all eight parameters are summarised in Table 4 assuming LV EDP of 10 mmHg and fibre angle ± 70°. It was observed that the values of each parameter were reduced with the increase in EF amongst the BVs. Moreover, the standard deviations of the parameters amongst the ventricles were not very high. A typical contour plot from the RSM and GA is shown in Fig. 5.

**Table 3:** Subject-specific four material parameter values of human myocardium with 10 mmHg LV EDP and helix angle = ± 70°

Subject	Passive material properties				EDV at 10 mmHg	Klotz curve resemblances
	*a* (kPa)	*b*	*Ka*	*Kb*		
BV1	0.080	6.00	6.10	2.80	83.01	0.09
BV2	0.092	4.80	6.80	3.10	118.89	0.20
BV3	0.089	4.76	6.98	3.30	98.08	0.15
BV4	0.060	4.45	7.20	3.40	138.03	0.16
BV5	0.048	4.38	7.30	3.30	101.04	0.12

**Table 4:** Subject-specific in vivo passive orthotropic eight material parameters’ value of human myocardium with 10 mmHg LV EDP and helix angle = ± 70°

Subject	Passive material properties							
	*a* (kPa)	*b*	*a*_*f*_ (kPa)	*b*_*f*_	*a*_*s*_ (kPa)	*b*_*s*_	*a*_*fs*_ (kPa)	*b*_*fs*_
BV1	0.080	6.00	2.951	5.893	0.492	3.393	0.070	3.929
BV2	0.092	4.800	2.647	5.323	0.441	3.065	0.063	3.548
BV3	0.089	4.760	2.579	5.000	0.430	2.879	0.061	3.333
BV4	0.060	4.450	2.500	4.853	0.417	2.794	0.059	3.235
BV5	0.048	4.380	2.466	5.000	0.411	2.879	0.058	3.333
Average	0.074	4.878	2.628	5.214	0.438	3.002	0.062	3.476
SD	0.019	0.653	0.193	0.417	0.032	0.240	0.005	0.278

**Fig. 6:**
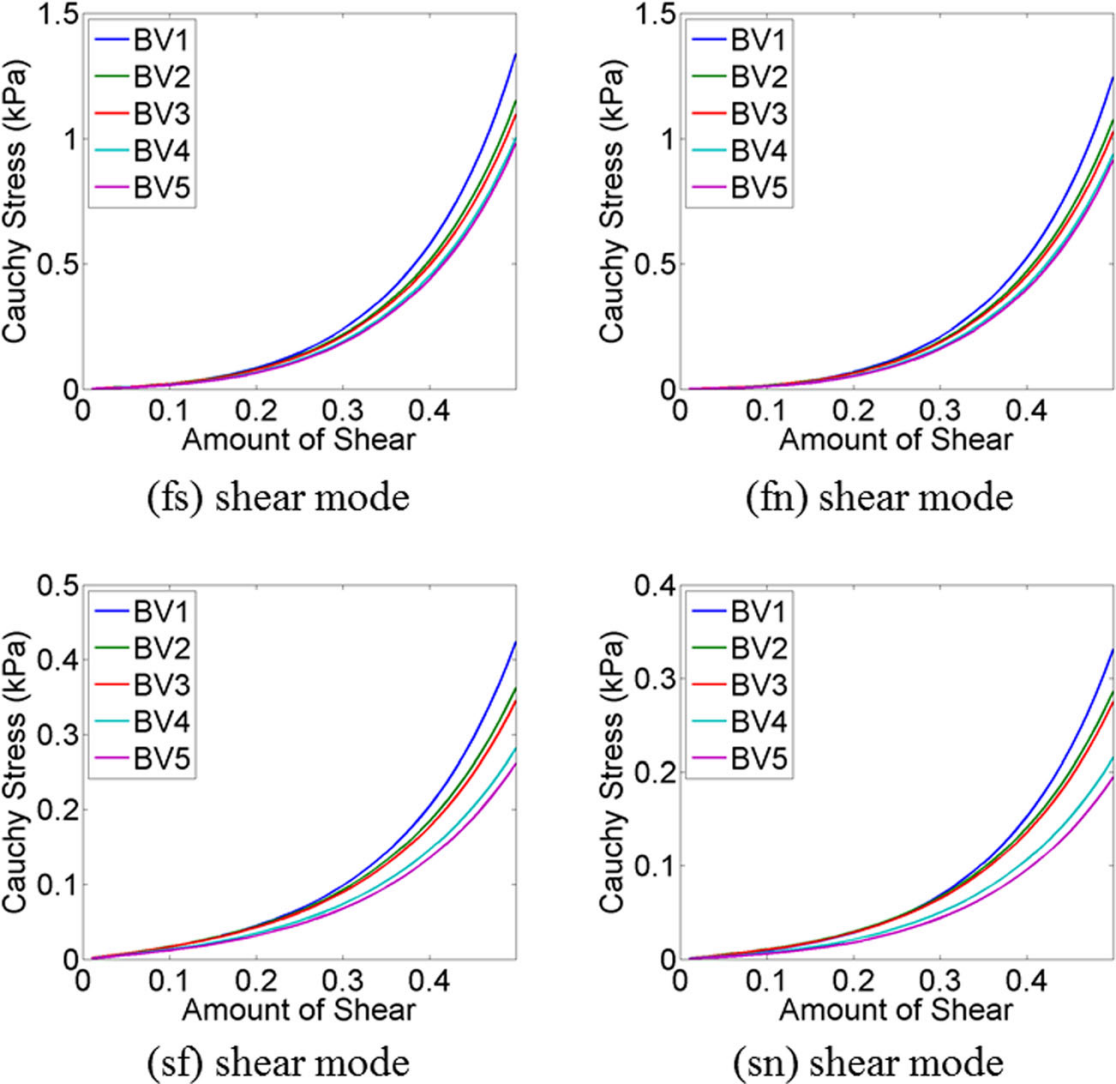
Compare shear stress–strain relationships for a cubic myocardial tissue under four different shear modes for five subjects (Table 4)

Shear stresses for four shear modes (σ^(*fs*)^, σ^(*fn*)^, σ^(*sf*)^, σ^(*sn*)^) were plotted (Fig. 6) using subject-specific values of the eight parameters, summarised in Table 4. It was observed that the predicted myofibre stress–strain relationships under different shear modes were reasonably similar for five subjects despite of the differences in predicted values. The similarities were more for shear in (*fs*) and (*fn*) plane compared to the shear in (*sf*) and (*sn*) plane.

### Sensitivity study

LV EDP was assumed 10 mmHg during the parameter estimation due to the unavailability of in vivo pressure data. Therefore, a sensitivity study was carried out to explore the changes in parameters if LV EDP was varied to 8, 12 and 15 mmHg. The estimated values of material parameters are depicted in Table 5. It was observed that the variation in parameter *b* was comparatively higher due to the change in EDP.

**Table 5:** Estimated values of the parameters with different end diastolic pressures (EDPs) for BV1

	Passive material properties							
	*a* (kPa)	*b*	*a*_*f*_ (kPa)	*b*_*f*_	*a*_*s*_ (kPa)	*b*_*s*_	*a*_*fs*_ (kPa)	*b*_*fs*_
8 mmHg	0.042	5.800	2.857	5.323	0.476	3.065	0.068	3.548
10 mmHg	0.080	6.00	2.951	5.893	0.492	3.393	0.070	3.929
12 mmHg	0.089	6.840	2.612	5.500	0.435	3.167	0.062	3.667
15 mmHg	0.120	8.200	3.600	7.174	0.600	4.130	0.085	4.783
Average	0.083	6.710	3.005	5.972	0.501	3.439	0.071	3.982
SD	0.032	1.091	0.421	0.836	0.070	0.481	0.010	0.557

Another sensitivity study was accomplished to investigate the effect of different fibre orientations on parameter estimation. BV1 mesh geometry and LV EDP of 10 mmHg were utilised for the study. Table 6 shows the values of eight parameters when fibre angle was ± 50°, ± 60°, ± 70° and ± 80°. It was observed that the effect of fibre orientation was comparatively grater on parameter *b.*

**Table 6:** Estimated values of the parameters with different fibre orientations with EDP = 10 mmHg for BV1

	Passive material properties							
	*a* (kPa)	*b*	*a*_*f*_ (kPa)	*b*_*f*_	*a*_*s*_ (kPa)	*b*_*s*_	*a*_*fs*_ (kPa)	*b*_*fs*_
Fibre 50	0.060	4.090	2.647	5.500	0.441	3.167	0.063	3.667
Fibre 60	0.086	4.900	2.769	5.500	0.462	3.167	0.066	3.667
Fibre 70	0.080	6.000	2.951	5.893	0.492	3.393	0.070	3.929
Fibre 80	0.114	8.010	2.951	5.500	0.492	3.167	0.070	3.667
Average	0.085	5.750	2.829	5.598	0.472	3.223	0.067	3.732
SD	0.022	1.698	0.149	0.196	0.025	0.113	0.004	0.131

**Fig. 7:**
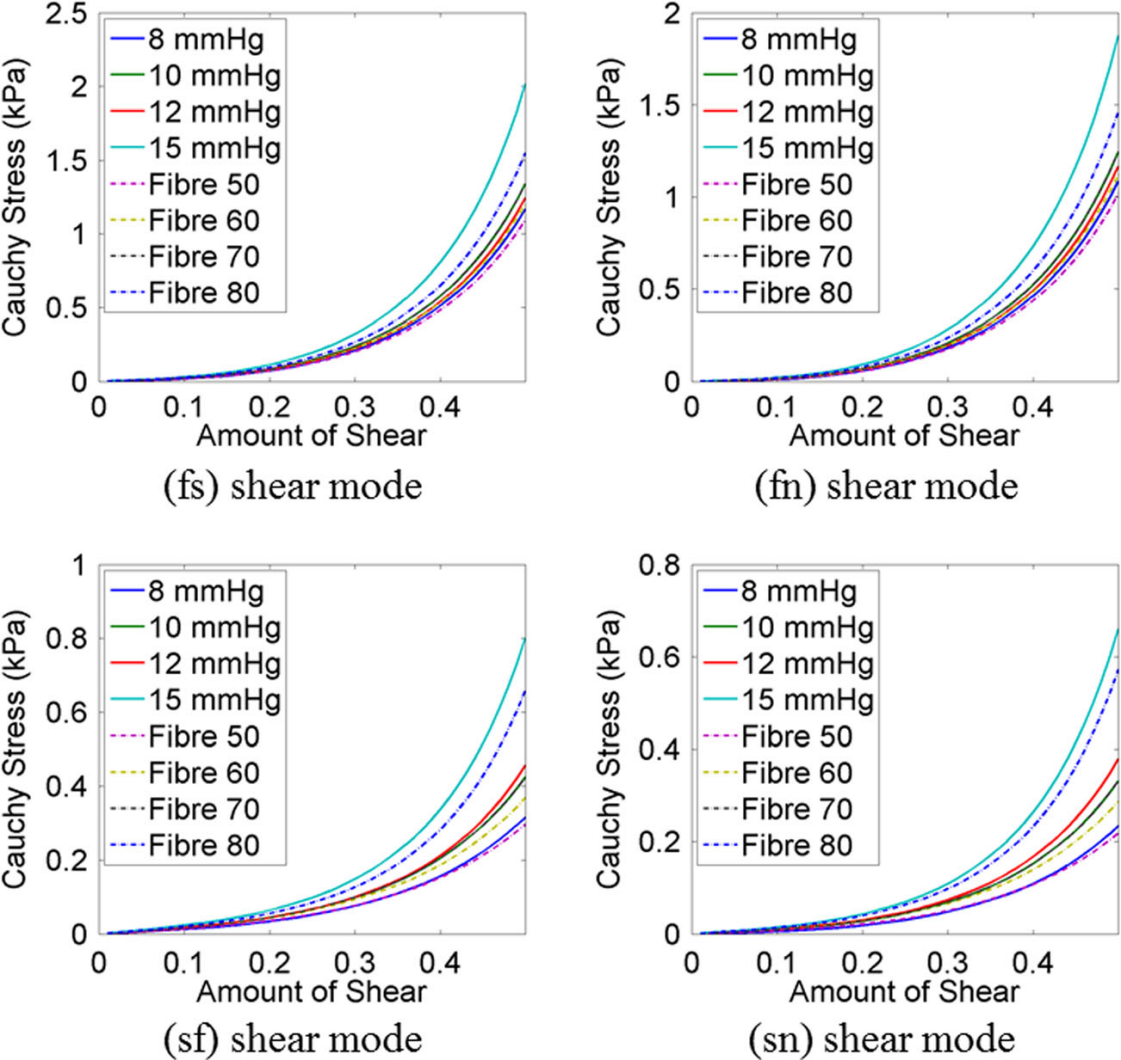
Compare shear stress–strain relationships for a cubic myocardial tissue under four different shear modes using the material parameter values, predicted with different EDP (Table 5) and with different fibre orientations (Table 6)

Shear stresses for four shear modes (σ^(*fs*)^, σ^(*fn*)^, σ^(*sf*)^, σ^(*sn*)^) were plotted (Fig. 7) using the material parameters, predicted with different EDP (Table 5) and with different fibre orientations (Table 6). It was noticed that the predicted σ^(*fs*)^ and σ^(*fn*)^ shear were quite similar except for 15 mmHg. When EDP was 15 mmHg, the myocardium became stiffer in fibre directions. The variation due to the fibre orientation was not very notable in (*fs*) and (*fn*) shear modes. However, the variation was higher for (*sf*) and (*sn*) shear modes although the predicted stress values were in the range from 0.4 to 0.8 kPa only.

**Fig. 8:**
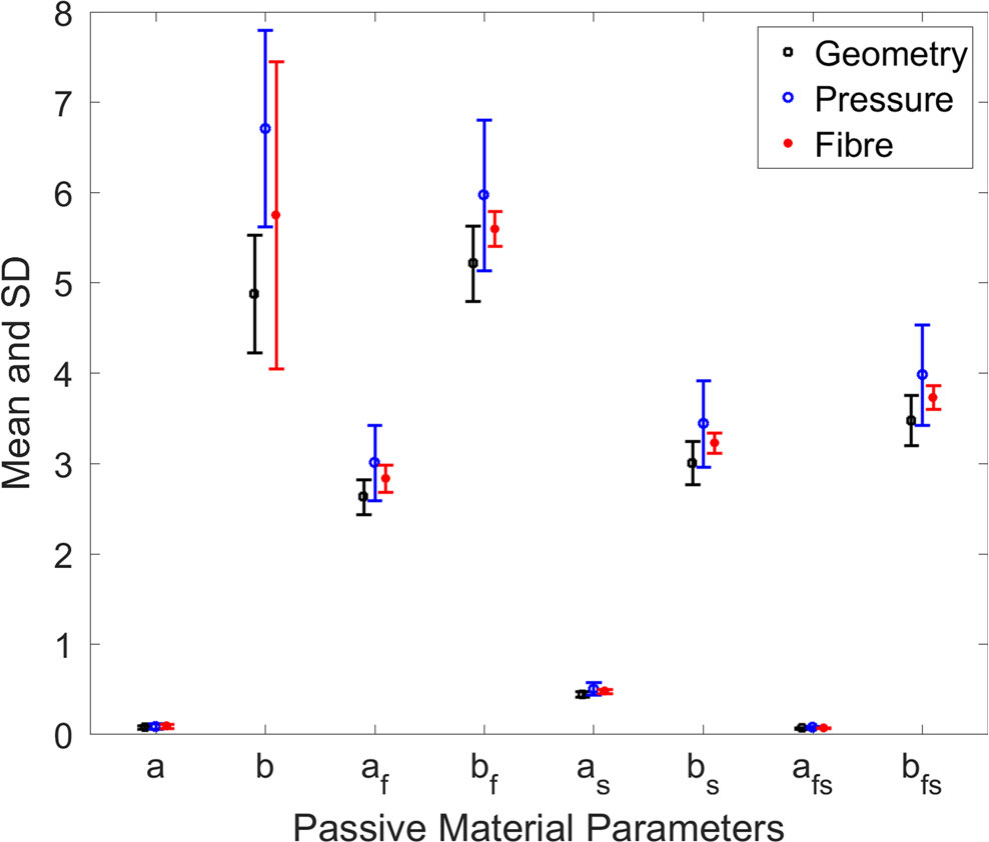
Effect on material parameters due to the changes in ventricular geometry, EDP and fibre orientation

Figure 8 shows variation in the material parameters due to the change in the following: (a) ventricular geometry (BV1, BV2, BV3, BV4 and BV5), (b) EDP (8, 10, 12 and 15 mmHg) for BV1 and (c) fibre orientation (± 50°, ± 60°, ± 70°, ± 80°) for BV1. It was observed that the effect of these variations on *a*, *a*_*s*_, and *a*_*f*s_ were negligible whereas the variation in *b* parameters was the highest. The other parameters *a*_*f*_, *b*_*f*_, *b*_*s*_ and *b*_*fs*_ experienced moderate variation. The effect of change in fibre orientation was the highest in parameter *b*. It was identified that the greater variation in material parameters occurred due to the change in EDP rather than the variation of ventricular geometries and fibre orientations.

### Compare with the state-of-the-art

**Fig. 9:**
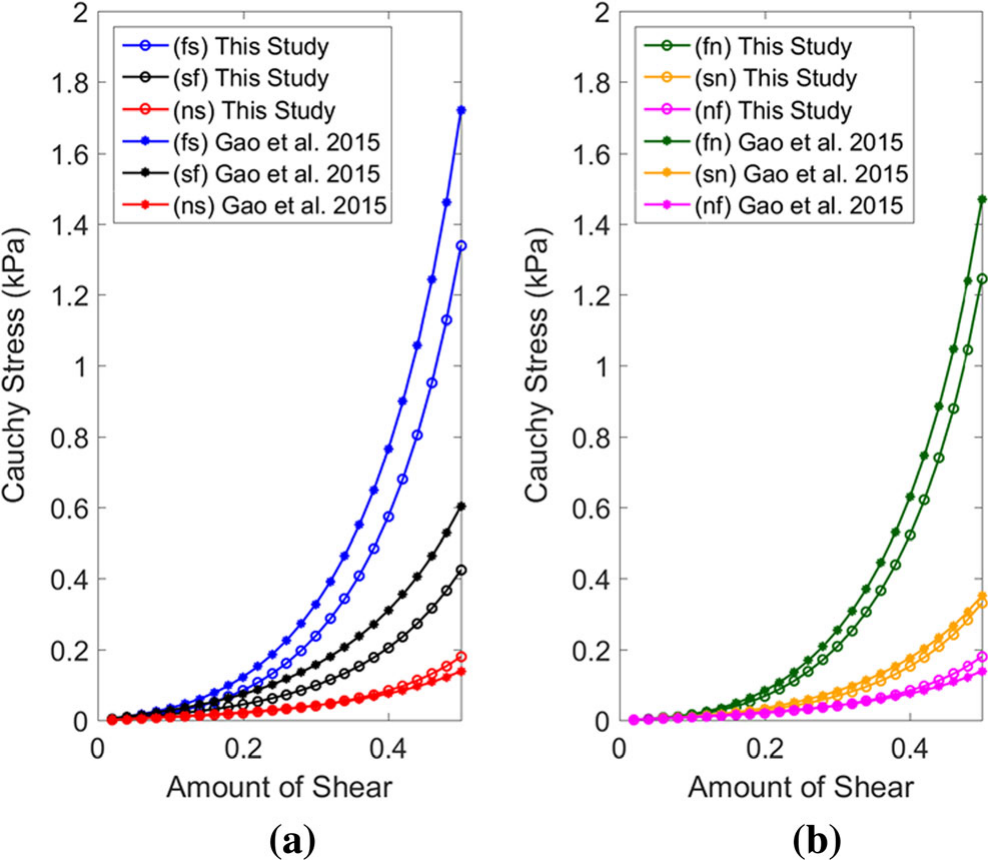
Comparison between shear stress-strain relationships for a cubic myocardial tissue under different shear modes using the material parameter values, predicted in this study and the values predicted by Gao et al. (2015). The parameters value identified with LV EDP of 10 mmHg was selected from both the studies. The parameters value for BV1, shown in Table 4, were used for this plot

The estimated values of the parameters in this study were further compared with the studies which inversely estimated human myocardium parameters. The shear stress–strain relations under six different shear modes of a cubic myocardium tissue were derived using the parameters identified in this study (for BV1) with the results from Gao, Li [12]. The parameters identified with LV EDP of 10 mmHg were selected from both the studies. Figure 9 shows that there is a good agreement between the predicted shear stress–strain relations, and therefore, between the estimated parameters values from both the studies. Clearly, it was observed that the shear stress–strain relations, measured from the traditional ex vivo experiment on excised human myocardium tissue [44], were overstiff. The *fs* and *fn* shear modes were in the range from 6 to 7 kPa from ex vivo measurement [44] whereas it was within the range from 1.2 to 1.8 kPa from in vivo when the amount of shear was 0.5. These discrepancies might be due to the tissue homoeostasis [8] in ex vivo conditions.

**Fig. 10:**
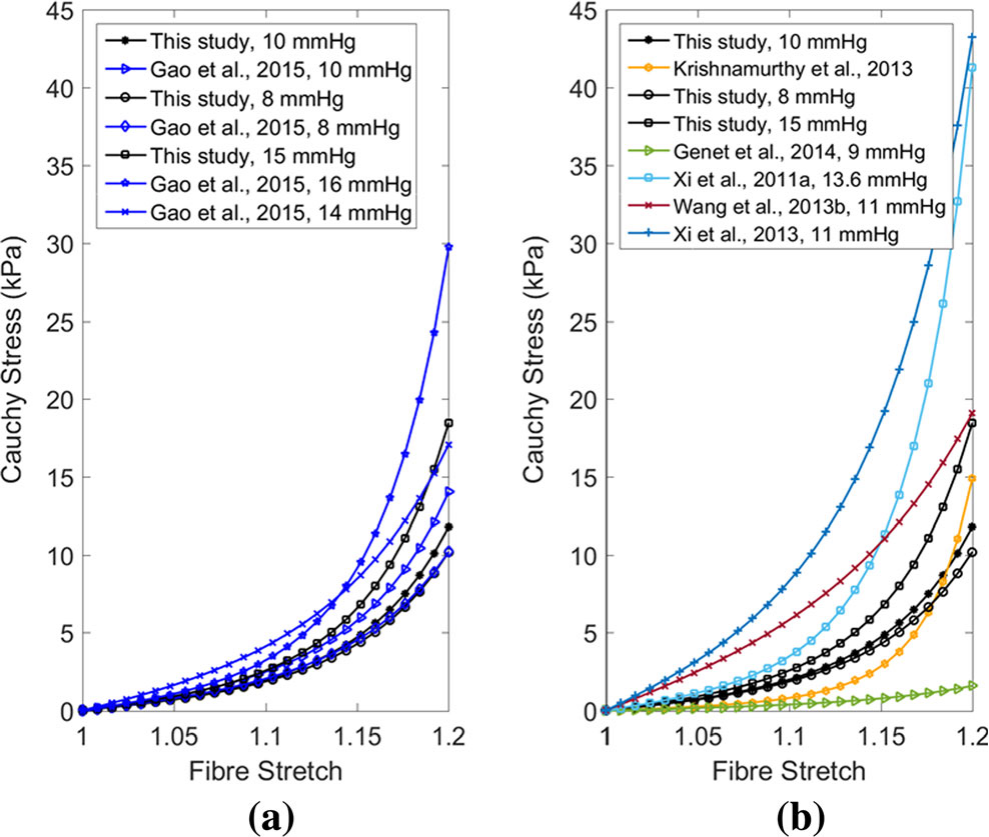
Comparison between stress–strain relationships for a cubic myocardial tissue under uniaxial stretch using the material parameter values, predicted in this study and in literature

Another study was carried out by comparing the stress–strain relation of a cubic myocardial tissue under uniaxial stretch test using the estimated parameters in this study with those from other studies, which inversely estimated human myocardial parameters. Xi, Lamata [62] estimated the values of four parameters for healthy human myocardium using transversely isotropic Fung-type law and LV EDP of 13.6 mmHg (1.81 kPa). Wang, Young [55] calibrated the pressure scaling parameter (C1) only of transversely isotropic Fung-type law for healthy human myocardium with LV EDP of 11 mmHg, and the values of other three parameters were taken from canine studies published in Wang, Ennis [54]. Krishnamurthy, Villongco [24] reported the values of four parameters (*a*, *b*, *a*_*f*_, *b*_*f*_) using transversely isotropic part of Holzapfel-Ogden model for human LV with heart failure. Genet, Lee [13] reported the passive material properties of human myocardium using transversely isotropic Fung-type law with 9 mmHg LV EDP. Furthermore, the parameter values estimated with different EDP in this study and by Gao, Li [12] were also used for uniaxial stress–strain relation comparison. Figure 10 summarises all the uniaxial stress–strain results using the respective constitutive parameters. Although, discrepancies existed amongst the plotted results, which were due to the subject variety (geometry, fibre orientation, EF) and different constitutive laws, the overall trend of the mechanical responses were reasonably similar.

### Differences in model predictions—pig vs human myocardium data

**Fig. 11:**
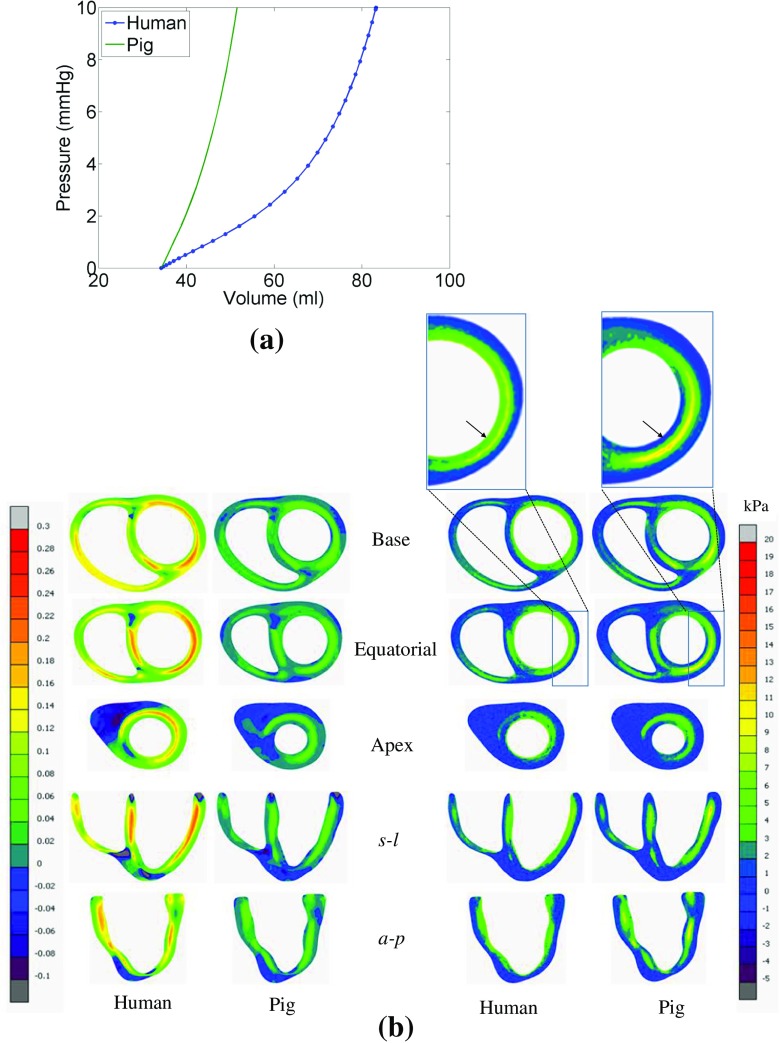
Comparison between the model predictions using the data set for pig and human myocardium. **a** Comparison between EDPVRs of LV. **b** Comparison between true fibre strain and fibre stress (Cauchy stress); details of the slice position were explained in Palit et al. (2015). The arrow sign in zoom in boxes show that the endocardium experiences high fibre stress for human ventricle whereas for pig, it starts after endocardium in posterior region of LV wall

It was observed that true fibre strain in LV wall is more for human data, because it is less stiff than pig myocardium, and therefore, inflated more at diastole (Fig. 11a). However, close inspection indicated that the fibre strain and stress distributions patterns were almost similar for both data sets, even though quantitative differences existed. For human data, lateral and posterior regions of endocardium were experienced high fibre stress, whereas for pig data, the same regions were experienced high stress but in the middle of the wall (not exactly in endocardium) (Fig. 11b). Details of the slice positions were described in Palit, Bhudia [35].

## Discussion

The biomechanical properties of myocardium, derived from ex vivo cadaver heart, might be different from in vivo properties due to tissue homeostasis. Therefore, estimation of in vivo passive orthotropic biomechanical properties of LV myocardium could provide improved insight of the physiology of the heart functionalities. This study introduced a novel method consisting of FE modelling, response surface method (RSM) and genetic algorithm (GA) to non-invasively estimate the Holzapfel-Ogden constitutive parameters using routinely used clinical data rather than using invasive and computationally expensive clinical data. However, the method is also capable of adding new subject-specific data (strain values, calculated from MRI tagging or personalised EDPVR, measured invasively) as a constraint to produce unique solutions. In addition, effects of end diastolic pressure, fibre structure and geometrical heterogeneity on estimated values of the parameters were also examined, and it was observed that the greater variation in material parameters occurred due to the change in EDP rather than the variation of ventricular geometries and fibre orientations. These estimated parameters of healthy human myocardium can be used directly in future studies.

The objective of the study was to estimate the subject-specific passive orthotropic properties of human myocardium using inverse optimisation method. This is a major challenge in the field of cardiac biomechanics [61] due to the following reasons: (a) limited in vivo clinical data due to the technical issues, invasive measurements and ethical issues, (b) highly non-linear nature of the inverse optimisation problem, (c) strong inter-correlations of the material parameters and (d) increase in design space of the optimisation problem due to the increase in number of parameters to incorporate fully orthotropic nature. Different constitutive laws have been developed by many researchers over the past to define myocardium characteristics [4, 5, 17, 18, 30, 45, 49]. Each law has corresponded to different sets of parameters and behaviours, and therefore, specific inverse optimisation approach was required. Structure-based, orthotropic Holzapfel-Ogden material law was used in this study to define myocardial characteristics as it has been gaining popularity in recent past over the other material laws (such as Fung-type transversely isotropic or orthotropic law) for heart modelling [12, 52]. However, unique estimation of eight material parameters from the limited in vivo clinical data is not possible due to the ill-posed nature of the inverse problem. Therefore, in this study, human myocardial stiffness was extracted by identifying the normal ranges of parameters with different conditions, rather than finding unique solutions.

Due to the insufficient clinical data, solving the inverse optimisation problem for fully orthotropic Hozapfel-Ogden law became challenging. To overcome this, various constraints were introduced to reduce the complexity of the problem. Inspired by the previous studies [13, 28, 60–62], the complexity of the problem was reduced by estimating a total of four parameters (*a*, *b*, *Ka*, *Kb*) instead of eight. The decision was based (i.e. reduction in number of independent parameters) on the shear test results of myocardium [9, 44]. It was observed from the experiment that the order of shear responses in six shear modes would follow as *σ*^(*fs*)^ > *σ*^(*fn*)^ > *σ*^(*sf*)^ > *σ*^(*sn*)^ > *σ*^(*nf*)^, *σ*^(*ns*)^ [9, 18]. Comparing the analytical expression of these six shear modes, it was concluded that only four parameters would be enough to maintain such characteristics. Besides, initial feasibility study was conducted to narrow down the ranges of four parameters so as to reduce the design space, and subsequently, to produce uniformly well-distributed Latin hypercube sample data.

Subject-specific LV EDP and fibre-orientations were not available due to invasive measurement and technical limitations [13, 26, 27, 52, 56]. Therefore, additional sensitivity studies were performed in order to identify the changes in material parameters due to the change in LV EDP and fibre orientations. Shear stress–strain relations under four different shear modes showed that the mechanical responses were similar, and within the physiological range even though the values of the parameters were changed due to the different EDP and fibre-orientations. These observations concurred excellently with the sensitivity study of Wang, Gao [52] using pig myocardium data and Holzapfel-Ogden model. One possible explanation for the same stress–strain relationship from different parameter values of the same constitutive law is that the law is designed in such a way that *I*_4*f*_ and *I*_4*s*_ terms have major contributions in stress prediction. The estimated parameters that influence these terms (*I*_4*f*_ and *I*_4*s*_) in the Holzapfel-Ogden constitutive law were relatively close for all the cases, and therefore, these parameter sets yielded similar mechanical responses.

Validation of the inversely estimated parameters for each human ventricle is not feasible as it is very challenging to perform mechanical tests on in vivo human hearts. Therefore, stress–strain relations of a cubic myocardium under simple shear and uniaxial stretch were compared with previous studies [12, 13, 24, 54, 60, 62]. It was observed that reasonably similar mechanical responses were predicted even with the differences existed in the used methods, material law, LV geometry, EDP and fibre orientations. Gao, Li [12] did not consider the effect of different fibre-orientation on material parameters estimation. However, it was shown that the passive inflation of LV increased with the increase in helix-angle [35]. Also, the distribution of fibre stress altered due the change in fibre structure [35]. In this study, the effect of different fibre orientations on estimated material parameters were also explored for the first time.

SSFP cine CMRI was used to construct subject-specific bi-ventricular geometry, and subsequently, the FE modelling of LV. Although MRI tagging is able to provide in vivo strain measurement, this requires additional scanning time, and subsequently, needs to perform complex image processing to calculate strain values. Besides, MRI tagging is not a routine clinical procedure whereas cine CMRI is performed routinely and readily available. LV strain can be measured from 2D cine CMRI as described by Gao, Li [12]. However, it has several limitations. These include difficulties in prediction of out-of-plane motion, and increase in uncertainties while estimating pixel-wise strain due to lack of motion tracking algorithm [12]. To overcome this, end diastolic pressure volume relation (EDPVR) of LV was used in the study instead of using strain calculation. However, subject-specific measurement of EDPVR requires invasive measurements which are not routinely performed in the clinical setting. Empirical Klotz curve was, therefore, incorporated to yield pressure-volume relation of LV. Klotz, Hay [23] reported that volume-normalised EDPVRs of LV have a common shape, irrespective of different species and diseased ventricles. Besides, comparing in vivo strain can only be possible at ED frame whereas pressure-normalise volume relation can be compared with each pressure point with empirical Klotz curve. The trade-off is between fewer data making the inverse problem more ill-posed compared to the requirement of more subject-specific, complex, invasive and time-consuming clinical data. Cine MRIs are routinely performed in the clinical setting only. Therefore, one of the major challenges in the study was to use standard clinical data to estimate human myocardial parameters. The estimated parameter values produced similar stress–strain results with those of other studies using MRI tagging and invasive measurements. However, in vivo strain data and subject-specific EDPVR, if available in the future, can easily be incorporated in the proposed method by adding new constraints while solving the optimisation.

One limitation of the study was the assumption of an initial stress-free state, which was present in all previous simulations of the heart based on in vivo images [12, 13, 34, 35, 47, 49, 57, 59]. Wang, Luo [53] reported that the effects of such initial (residual) stresses are relatively small in late diastole when pressure is higher. In contrast, a recent study observed measurable effect (reduce the LV stiffness by 40% during passive filling) of pre-stress during diastole [14]. Therefore, it is still an open question and future studies will be carried out to consider physiological pre-stress condition to identify the effect of residual stress on parameter estimation.

## Conclusions

In this study, subject-specific in vivo passive material properties of human myocardium were estimated using inverse optimisation procedure. MRI measured EDV and empirical Klotz relation were used to scale the material parameters of the Holzapfel-Ogden model for five healthy human ventricles. Anatomically realistic subject-specific models of five human bi-ventricles (BVs), constructed from CMRI, that employed rule-based fibre-sheet orientation and a structure-based orthotropic constitutive law (Holzapfel-Ogden model) were used to simulate the passive diastolic mechanics. This study introduced a novel method consisting of FE modelling, response surface method (RSM) and genetic algorithm (GA) to non-invasively estimate the Holzapfel-Ogden constitutive parameters using routinely used clinical data rather than using invasive and computationally expensive clinical data. Due to the limited clinical data, two different sensitivity studies were accomplished to identify the changes in parameters with the change in EDP and fibre orientations. Comparison of simple shear and uniaxial stress–strain relations with other studies, which inversely estimated human myocardial parameters based on different constitutive laws and EDP, showed that the estimated material parameters in this study generated similar stress–strain predictions. The study provided a wide range of parameter values due to the change in geometry, EDP and fibre orientations. These information could be useful for future computational study to identify the normal ranges of myocardial wall stress and strain during cardiac cycle.

## Appendix 1

**Table 7:** Typical Scanning parameters for SSFP CMRI

	Short axis	Long axis
FOV	User adjustable (depends on patient size) but typically ~ 37 cm square	User adjustable (depends on patient size) but typically ~ 37 cm square
Acquisition matrix	192 × 200 pixels	200 × 200 pixels
Reconstructed image matrix	512 × 512 pixels	512 × 512 pixels
Flip angle	50°	50°
Bandwidth	325.5 Hz/pixel	325.5 Hz/pixel
TR	4.4 ms	3.5 ms
TE	1.6 ms	1.5 ms
Slice thickness	8 mm	8 mm
Slice gap	0	0
Frames per cardiac cycle	30	30

**Table 8:** Demographic information

	Sex	Age (years)
BV1	Female	39
BV2	Male	54
BV3	Male	28
BV4	Male	38
BV5	Male	36

## Appendix 2. Structure-based constitutive law for passive myocardium—Holzapfel-Ogden model

The strain energy function developed by Holzapfel and Ogden (2009) was extended by Göktepe et al. (2011) using decoupled volumetric-isochoric formulation of finite elasticity. The deformation gradient (**F**) is multiplicatively decomposed into a volumetric part ***F***_**vol**_ and an isochoric part $\overline{\mathbf{F}}$ as,

B.1 $$ \mathbf{F}=\overline{\mathbf{F}}\;{\mathbf{F}}_{\mathbf{vol}}\kern1.08em \mathrm{with}\kern0.6em {\mathbf{F}}_{\mathbf{vol}}={\mathbf{J}}^{1/3}\;\mathbf{I}\kern0.72em \mathrm{and}\kern0.6em \overline{\mathbf{F}}={J}^{-1/3}\;\mathbf{F} $$

such that *J* = det (**F**_**vol**_) and $\det \left(\overline{\mathbf{F}}\right)=1$. The right Cauchy-Green tensor is defined as$\mathbf{C}={J}^{2/3}\;\overline{\mathbf{C}}$, where $\overline{\mathbf{C}} = {\overline{\mathbf{F}}}^{\mathbf{T}}\;\overline{\mathbf{F}}$denotes the modified tensor quantities. The myocardium tissue is an orthotropic material with the fibre, sheet and sheet-normal directions denoted by **f**_**0**_, **s**_**0**_, **n**_**0**_, respectively, in the Lagrangian framework. The strain energy function Ψ per unit reference volume is additively decomposed into volumetric Ψ_*vol*_(*J*) and isochoric $\overline{\Psi}$ parts,

B.2 $$ \Psi ={\Psi}_{vol}(J)+\overline{\Psi}\left({\overline{I}}_1,{\overline{I}}_{4f},{\overline{I}}_{4s}\;{\overline{I}}_{8 fs}\right) $$

where Ψ_*vol*_(*J*)and $\overline{\Psi}$ are given scalar-valued function of *J* and the isochoric invariants ${\overline{\boldsymbol{I}}}_1,{\overline{\boldsymbol{I}}}_{4\boldsymbol{f}},{\overline{\boldsymbol{I}}}_{4\boldsymbol{s}},{\overline{\boldsymbol{I}}}_{8\boldsymbol{fs}}$, respectively, and

B.3 $$ {\overline{I}}_1= Tr\left(\overline{\mathbf{C}}\right),\kern0.96em {\overline{I}}_{4f}={\mathbf{f}}_{\mathbf{0}}\cdot \left(\overline{\mathbf{C}}\;{\mathbf{f}}_{\mathbf{0}}\right),\operatorname{}{\overline{I}}_{4s}={\mathbf{s}}_{\mathbf{0}}\cdot \left(\overline{\mathbf{C}}\;{\mathbf{s}}_{\mathbf{0}}\right),\operatorname{}\kern0.6em {\overline{I}}_{8 fs}={\mathbf{f}}_{\mathbf{0}}\cdot \left(\overline{\mathbf{C}}\;{\mathbf{s}}_{\mathbf{0}}\right) $$

Ψ_*vol*_(*J*) is defined as

B.4 $$ {\Psi}_{vol}=K\left(\frac{J^2-1}{2}-\mathrm{lnJ}\right) $$

where *K* is the user-specified bulk-modulus and serves as a penalty parameter to incorporate material incompressibility. $\overline{\Psi}$ consists of an isotropic part $\left({\overline{\Psi}}_{iso}\right)$ and an anisotropic part $\left({\overline{\Psi}}_{aniso}\right)$. ${\overline{\Psi}}_{iso}$ depends only on ${\overline{I}}_1$ and represents the contribution of an isotropic ground matrix of the myocardial material such as

B.5 $$ {\overline{\Psi}}_{iso}=\frac{a}{2b}\;\exp \left[b\left({\overline{I}}_1-3\right)\right] $$

${\overline{\Psi}}_{aniso}$ corresponds to the contributions from the myocytes and families of collagen fibre embedded within the ground matrix. The function is described as

B.6 $$ {\overline{\Psi}}_{aniso}=\sum \limits_{i=f,s}\frac{a_i}{2{b}_i}\;\left\{\exp \left[{b}_i{\left({\overline{I}}_{4i}-1\right)}^2\right]-1\right\}+\frac{a_{fs}}{2{b}_{fs}}\left\{\exp \left[{b}_{fs}{\left({\overline{I}}_{8 fs}\right)}^2\right]-1\right\} $$

The collagen fibres do not support compression. This condition is imposed by including the terms containing the directionally dependent invariants (I_4 f and I_4s) in strain energy function only when I_4 f > 1 or I_4s > 1 (Holzapfel and Ogden, 2009). There are total eight non-negative material parameters in the strain energy function (*a*, *b*, *a*_f_, *b*_f_, *a*_s_, *b*_s_, *a*_8fs_, *b*_8fs_) along with an extra bulk modulus. Hence, the total strain energy function becomes

B.7 $$ {\displaystyle \begin{array}{l}\Psi =K\left(\frac{J^2-1}{2}-\mathrm{lnJ}\right)+\frac{a}{2b}\;\exp \left[b\left({\overline{I}}_1-3\right)\right]\;\\ {}\kern1.08em +\sum \limits_{i=f,s}\frac{a_i}{2{b}_i}\;\left\{\exp \left[{b}_i{\left({\overline{I}}_{4i}-1\right)}^2\right]-1\right\}+\frac{a_{fs}}{2{b}_{fs}}\left\{\exp \left[{b}_{fs}{\left({\overline{I}}_{8 fs}\right)}^2\right]-1\right\}\end{array}} $$

The second Piola-Kirchhoff stress tensor *S* = 2*∂ψ*/*∂****C*** is also divided into a purely volumetric part (**S**_**vol**_) and a purely isochoric part $\left(\overline{\mathbf{S}}\right)$, such as $\boldsymbol{S}={\boldsymbol{S}}_{\mathrm{vol}}+\overline{\boldsymbol{S}}$. The volumetric part is

B.8 $$ {\mathbf{S}}_{\mathbf{vol}}=J{p}_h{C}^{-1}\operatorname{} where\kern0.24em {p}_h=\frac{d{\Psi}_{vol}}{dJ}=K\left(\frac{J^2-1}{J}\right) $$

and the isochoric part is

B.9 $$ \overline{\mathbf{S}}=2{J}^{-2/3}\;\left[{\overline{\Psi}}_1\; DEV(I)+{\overline{\Psi}}_{4f}\; DEV\left({\mathbf{f}}_{\mathbf{0}}\otimes {\mathbf{f}}_{\mathbf{f}}\right)+{\overline{\Psi}}_{4s}\; DEV\left({\mathbf{s}}_{\mathbf{0}}\otimes {\mathbf{s}}_{\mathbf{0}}\right)+\frac{1}{2}\;{\overline{\Psi}}_{8 fs}\; DEV\;\left({\mathbf{f}}_{\mathbf{0}}\otimes {\mathbf{s}}_{\mathbf{0}}+{\mathbf{s}}_{\mathbf{0}}\otimes {\mathbf{f}}_{\mathbf{0}}\right)\right] $$

where *I* is identity tensor, and

B.10 $$ DEV\left(\bullet \right)=\left(\bullet \right)-\frac{1}{3}\;\left[\left(\bullet \right):\mathbf{C}\right]\;{\mathbf{C}}^{-1} $$

is the deviatoric operator in the Lagrangian description so that $DEV\left(\partial \overline{\Psi}/\partial \overline{C}\right):C=0$ and

B.11 $$ {\overline{\Psi}}_1=\frac{\partial \overline{\Psi}}{\partial {\overline{I}}_1}=\frac{a}{2}\;\exp \left[b\;\left({\overline{I}}_1-3\right)\right], $$

B.12 $$ {\overline{\Psi}}_{4i}=\frac{\partial \overline{\Psi}}{\partial {\overline{I}}_{4i}}={a}_i\;\left({\overline{I}}_{4i}-1\right)\;\exp\;\left[{b}_i\;{\left({\overline{I}}_{4i}-1\right)}^2\right],\kern0.36em \operatorname{}i=f,s, $$

B.13 $$ {\overline{\Psi}}_{8 fs}=\frac{\partial \overline{\Psi}}{\partial {\overline{I}}_{8 fs}}={a}_{fs}{\overline{I}}_{8 fs}\exp\;\left({b}_{fs}{{\overline{I}}_{8 fs}}^2\right) $$
